# Supplementary material for: Are carbon emissions trading and green financial instruments synergistic? -Comprehensive quantitative research based on content analysis
Source: PLoS One. 2024 Mar 7;19(3):e0298601. doi: 10.1371/journal.pone.0298601 (PMC10919601; doi:10.1371/journal.pone.0298601)
Supplement: S1 Data — (ZIP) [file pone.0298601.s001.zip › Data/地级市Z_F工作报告.zip!/░▓╗╒/┴∙░▓╩╨/└┤╫╘╣1⁄2╓┌║┼í╛┬φ┐╦╩2╛▌═°í┐_2008.docx]

各位代表：
　　现在，我代表市人民政府，向大会作政府工作报告，请予审议，并请市政协委员和其他列席人员提出意见。

**一、2007年成绩显著，
经济社会发展跨上新台阶**

　　2007年，在省委、省政府和市委的坚强领导下，全市人民以党的十七大胜利召开为动力，认真贯彻科学发展观，团结一心，拼搏奋进，战胜了淮河流域特大洪涝灾害，经济社会发展保持了增长加快、效益提高、活力增强、民生改善的良好态势，圆满完成了市二届人大三次会议确定的目标任务。
　　国民经济加快发展。初步核算，生产总值突破400亿元，达439.8亿元，增长15%以上；人均971美元，比上年增加220美元。财政收入32.9亿元，增长31%。农业总产值220.6亿元，增长11%。规模工业增加值95.2亿元，增长42.5%，增幅保持全省前列；1-11月效益综合指数166.1，比上年末提高20.5个百分点。社会消费品零售总额161亿元，增长18%。年末金融存款余额425.9亿元、贷款余额264.8亿元，分别增长18.2%、16%，比年初增加65.6亿元、36.5亿元，贷款增量首次跃居全省第3位。
　　经济结构逐步调整。三次产业结构调整为25.5：36.1：38.4。农业基础地位得到加强，粮食总产突破400万吨大关，再创历史新高，霍邱县连续三年获全国粮食生产先进县标兵；畜牧、水产业全面增长，蔬菜业连续四年居省产业化三十强之首。工业支撑明显增强，对经济增长贡献率达48%；产值超亿元企业81户，其中应流集团、大昌矿业和迎驾集团、庆发集团达到和逼近15亿元，新增规模企业130户；省级高新技术企业和产品分别达25家和31个，市科技创业服务中心建成运行。新兴三产加快发展，旅游收入19亿元，增长28%，大别山（六安）国家地质公园开园，天堂寨景区争创4A成功，张家店战役纪念馆建成开馆；金融生态明显改善，贷款不良率下降5.7个百分点；银行业金融机构盈利3.11亿元，是上年的2.9倍。环保专项行动和节能减排扎实开展，单位生产总值能耗下降4%，主要污染物排放量下降2.08%。
　　新农村建设扎实推进。“百村十镇”示范工程深入实施，50个重点镇建设全面加强。十大基地建设步伐加快，农业产业化龙头组织达850个，销售收入亿元以上企业15家，新获“三品”认证50个。农村科技发展格局优化，创建专家大院和选派特派员达34个和328名。农村公路村村通完成投资3.5亿元，新建1650公里，金寨县被评为全国农村公路建设先进单位。农业综合开发扎实推进，江淮分水岭综合治理开发十年成果丰硕，农田水利基本建设和土地整理成绩突出，耕地保护得到加强，完成8处省级、3处市级地质灾害点治理和37座病险水库除险加固。建成“万村千乡”农家店及配送中心603个，圆满完成三年任务，“新网工程”加快推进。解决了28万农村人口饮水安全问题。培训劳动力8万人。惠民资金管理“一线实”和农民“互助资金”试点取得积极成效，大中型水库移民后期扶持资金兑现到位。发放各类财政惠农补贴10.2亿元。
　　发展条件进一步改善。完成固定资产投资201.3亿元，增长27.5%；实施“568”计划项目287个，完成投资130亿元。应流3万吨铸件、草楼铁矿、大昌120万吨球团一期、庆发日产600吨大米加工、华晶高吸水性树脂、星星轻纺大麻汽爆和安踏服装、三希皮革一期、长江精工3万吨重钢、强大齿轮技改、佳瑞粉末冶金等项目建成投产或即将投产。8个省级开发区和工业园区新增建成面积5平方公里，完成基础设施投入5亿元，六安开发区在全省同类开发区综合经济运行评价中前进3个位次，霍山开发区被评为省首届投资环境十佳开发区。合六叶高速公路顺利通车，合武铁路和合武、合淮阜高速公路六安段建设加快推进。周集港一期工程建成运营，正南淮堤加固、淠河应急加固等工程全面建成，白莲崖水库、安丰塘除险加固、淮干三大泵站等工程加快实施。中心城市建设成绩突出，完成重点工程投资9.4亿元，建成区面积达44.2平方公里。市老年活动中心、大雁河排涝站、新河东西路部分路段改造、10条便民道路等重点工程相继竣工，旧城改造加快推进；城市管理百日会战收效明显，绿化、美化、亮化工程全面实施，市容市貌进一步改观，土地收储和融资平台建设成绩突出。城镇化进程加快，城镇化率达33.5%。 
　　改革开放深入推进。市属国有商贸物资流通企业改制基本完成，水利、交通、建筑等系统体制改革启动，农村综合改革、集体林权制度改革积极推进，乡镇政务服务中心达165个。招商到位内资95亿元、外资7594万美元，分别增长29%、16.3%。世界500强沃尔玛入驻六安。预计进出口总额2.3亿美元，增长15%。新增私营企业1.3万户，非公有制经济比重达58%以上。我市和霍山县被安徽省浙江商会评为最佳服务市、县，金寨县、舒城县获2007国际徽商年会（无锡）最具投资潜力奖。
　　人民生活较大改善。城镇居民人均可支配收入10459元，农民人均纯收入3058元，分别增长16.9%和21.3%。年末居民储蓄存款余额279.5亿元，增长13%，比年初增加32.2亿元。十二项民生工程全面实施，到位资金7.9亿元。将18.6万人纳入农村低保范围，为1.3万名城镇未参保集体企业退休人员发放生活费，城镇居民基本医疗参保27.3万人。新增就业2.7万人，其中再就业1.5万人，消除零就业家庭1035户，城镇登记失业率控制为3.8%。五大社会保险加快推进，养老保险市级统筹实行，被征地农民养老保险和生育保险全面实施，廉租住房、经济适用住房建设启动，安置房建设稳步推进，市住房公积金归集余额突破10亿元。加大扶贫整村推进力度，脱贫186个村、10万人。积极开展生产救灾和灾后重建，下拨救灾资金6320万元，筹集灾民建房扶持资金3903万元，兑现行蓄洪区补偿资金2175万元。
　　社会建设全面加强。教育教学质量不断提高，义务教育经费保障机制改革稳步推进，75.5万中小学生受益，城区义务教育学校改扩建工程启动，高中教育快速发展，示范高中水平进一步提升，消除农村中小学D级危房15万平方米。城乡卫生服务体系建设切实加强，新建城市社区卫生服务机构48家，改造乡镇卫生院32个，市人民医院门诊大楼投入运行，市中医院创“三甲”成功，新型农村合作医疗普及实施，参合农民478万人，参合率84.3%。农村电影2131工程、送戏下乡、广播电视村村通和农家书屋建设全面推进，电视转星调整顺利完成，网吧监控平台建成启用，双墩一号汉墓保护开发取得积极进展，寿县获全国文物工作先进县。全民健身活动蓬勃开展，市第七届老运会成功举办。低生育水平持续稳定，出生人口性别比偏高趋势逐步扭转，流动人口计划生育管理和服务得到加强，人口出生率控制为12.3‰。生态市建设成效明显，霍山县、舒城县分别获全国绿色小康县、全国首批绿化模范县。食品安全与产品质量、交通、消防等专项整治认真开展，安全生产形势、信访秩序总体好转，“平安六安”建设进一步深化，社会治安防控体系逐步完善。全国双拥模范城争创成功，六安军分区被评为全国拥政爱民模范单位，国防动员、民兵预备役工作进一步加强。各类精神文明创建活动蓬勃开展，“五五”普法全面推进，妇女、儿童、老年人、残疾人权益得到保障，民族宗教工作水平不断提高，外事侨务、人事编制、人防气象、统计物价、档案方志、地震文物、对台事务、邮政通信、新闻出版、保险证券、供电烟草、砂石管理等工作都取得了新的成绩。
　　行政能力切实提高。市政府自觉接受人大法律监督和政协民主监督，办结人大议案3件、代表建议127件和政协提案267件。行政执法依据清理完成，认定行政执法主体63个，组建了市城市管理行政执法局。行政执法监督力度加大，执法行为进一步规范。“三型机关”创建深入推进，行政效能明显提高。认真落实重大决策咨询、听证、公示等制度，加快推进政务公开，市政务服务中心效能建设大力推进、服务水平全面提升。市政府网站全面改版，无纸化办公启动，在线访谈、市长热线和政风行风热线受到欢迎。加强行政监察和审计监督，组建了市招投标监督管理局。经济发展环境保护和纠风工作扎实开展，案件查处力度加大。应急管理体系建设全面推进，处置突发事件能力不断增强。
　　各位代表！过去的一年，我们在遭受严重洪涝灾害的情况下迈出了六安跨越发展的坚实步伐，令人振奋，催人奋进。这是市委正确领导的结果，是市人大、市政协监督支持的结果，是全市人民团结奋斗、开拓进取的结果。在此，我谨代表市人民政府，向奋战在各条战线上的全市人民，向市人大代表、政协委员和各民主党派、工商联、各人民团体和其他各界人士，向中央、省属驻六安单位和解放军指战员、武警官兵、政法干警，向在我市创业的境内外投资者、建设者和所有关心、支持六安建设发展的朋友，表示崇高的敬意和衷心的感谢！
　　当前发展中存在的主要问题是，经济总量不大，产业集聚度不高，骨干企业支撑和拉动能力有待增强；投入总量不足，大项目不多，土地、资金、人才、环境等约束性因素增多，持续发展能力有待提高；社会保障能力不强，居民消费价格上涨较快，改善民生还有不少问题有待解决；政府职能转变还没到位，行政效能和服务水平有待提升。对这些问题，我们将高度重视，采取有力措施，认真加以解决。

**二、认真贯彻落实党的十七大精神，
奋力加快全面建设小康六安步伐**

　　2008年是全面贯彻落实党的十七大提出的战略部署的第一年。按照市委二届九次全会的部署，今年工作的总体要求是，全面贯彻党的十七大和中央、全省经济工作会议精神，以邓小平理论和“三个代表”重要思想为指导，以科学发展观为统领，以全面建设小康六安为主题，以转变经济发展方式为主线，深化改革，扩大开放，扎实推进工业化、城镇化、农业产业化，扎实推进新农村建设，扎实推进民生工程，扎实推进和谐社会建设，实现经济社会又好又快发展。
　　主要预期目标：生产总值增长12%以上，财政收入增长20%以上，全社会固定资产投资增长25%以上，社会消费品零售总额增长15%，进出口总额增长15%，招商到位内资和外资分别增长20%、15%，城镇居民人均可支配收入和农民人均纯收入分别增长12%、8%，城镇新增就业2.35万人以上，城镇登记失业率控制在4.2%以内，人口出生率控制在13.6‰以内，单位生产总值能耗下降4.3%，化学需氧量和二氧化硫排放量分别下降2.3%、5%。
　　党的十七大提出了全面建设小康社会新要求，其中人均生产总值到2020年比2000年翻两番。我市2020年要达到全国人均5000美元的水平，今后13年生产总值平均增幅必须达到13%以上。为此，我们要牢牢抓住发展第一要务，进一步增强贯彻科学发展观的坚定性、主动性和创造性，进一步弘扬老区人民勇于拼搏、敢于胜利的传统，进一步激发跨越的勇气和崛起的劲头，坚定信念，团结拼搏，力争与全国同步跨入全面小康。

**三、着力转变发展方式，
促进经济又好又快发展**

　　在经济持续较好较快增长的基础上，进一步加快结构调整，转变发展方式，培育新的增长点，积极有为地贯彻落实中央宏观调控措施，不断增强发展后劲和综合竞争力。
　　（一）坚持工业化核心战略，加快工业强市步伐。深入开展“发展工业学霍山”活动，着力推进科技创新和管理创新，加快工业发展融资平台建设，培育百亿元产业，打造十亿元企业，壮大亿元企业，催生规模企业，确保规模工业增加值增长25%以上、利税增长18%以上，工业固定资产投资增长40%以上。
　　培育优势产业和产业集群。紧扣省千亿元产业发展规划和省会经济圈现代产业体系，依托应流集团、大昌矿业、迎驾集团、庆发集团、金安钢铁、世林集团、华润啤酒、天业集团、江淮电机、江汽齿轮、强大齿轮、星星轻纺、一隆羽绒、海洋羽绒、六安电厂、精工钢构、华源制药、建来化工等骨干企业，加强钢铁冶金、农副产品深加工、汽车零部件及机械制造、纺织服装、电力能源、建筑建材、医药化工等重点产业基地建设。坚持“四个定期”，实施“五个倾斜”，促进骨干企业快速成长。培育超亿元企业达100户以上，其中应流集团、大昌矿业产值分别力争超30亿元、20亿元，另有5户以上超10亿元，力争钢铁产业率先过百亿。设立上市办，支持企业开展资本运作，确保应流集团香港上市，力争1-2家境内上市。依托园区、优势产业和骨干企业完善产业链条，促进一批专业化水平高、配套能力强的中小企业发展。扩大企业创新基金规模，新增规模企业100户以上。
　　增强企业创新能力。引导和支持骨干企业与科研机构、大专院校开展研发机构共建、人才委培引进、技术难题攻关、科技成果转让、知识产权入股等多形式、深层次合作，促进科技成果转化。设立种子资金，完善市科技创业服务中心功能，逐步建立各级公用技术服务平台。支持企业创建省级以上技术中心5个左右，重点实施10个传统产业升级改造和20个技术创新及一批信息技术应用项目。大力发展高新技术产业，新产品产值率达30%以上。
　　推进开发园区建设。抓紧做好开发园区规划的完善和修编工作，健全激励约束机制，创新融资渠道，完善基础设施和公共服务体系，力争在产业结构、企业档次、园区配套、服务效能等方面全面提升。市开发区要在招商安商亲商上下更大气力，力争到位内资和外资分别达12亿元和1500万美元以上，生产总值和财政收入分别增长30%和40%以上。各县区园区引资到位6亿元以上，引进千万元以上项目15个以上，并要在3000万、5000万和亿元项目上多着力。严格执行开发区单位面积投资强度标准，加大闲置土地清理力度。
　　（二）加快发展农业和农村经济，扎实推进新农村建设。坚持把“三农”工作作为重中之重，以发展现代农业为重点，促进农业稳定增效、农民持续增收、农村更加和谐。
　　发展现代农业。大力调整农业生产结构，转变农业增长方式，推进十大基地建设，实施大型国家商品粮生产基地项目，创建全省优质粮食产业工程示范区，建设中央储备粮粮源基地，促进粮食生产增量提质，冲刺百亿斤大关。落实扶持政策，促进油料生产。认真实施省农业产业化提升行动，新培育1家国家级、10家以上省级和新认定20家以上市级龙头企业，发展农民专业合作组织100个。推进农业标准化、规模化、机械化，加强农产品“三品”认证，发展品牌农业。落实好省生猪产业发展“462”计划和渔业三进工程，新建畜禽标准化养殖小区100个、水产标准化养殖小区20个，新增存栏奶牛3000头。高度重视“菜篮子”产品生产，保持市场供应和价格稳定。抓好农产品展示展销活动，加大名优农产品宣传和招商力度。加强农资市场监管，健全农产品质量安全保证体系。
　　加强农村基础设施和公共服务体系建设。认真实施新农村建设规划，加快推进“百村十镇”示范工程，引导农民建房向规划点集中。新建农村公路村村通1600公里，新改建通乡油路380公里。深入推进江淮分水岭综合治理开发，加快淠史杭灌区续建配套和节水改造，完成80座小型水库除险加固，改善以水利为重点的农业生产条件。加快农村安全饮水、清洁能源工程，解决37万农村人口饮水安全问题，新增农村沼气用户5000户。推动农村科技工作重心下移，强化农业科技推广体系建设，做好科技特派员创业行动和林业特派员试点工作。加强新农村民风建设，加大农村环境卫生整治力度，营造整洁、优美的人文人居环境。
　　构建农民增收长效机制。引导社会、民间、外资资本进入农村经济各个领域，积极争取省政策性农业保险试点。大力发展劳务经济，推进“阳光工程”，调动多方积极性，培训8万人以上。切实保障农民工合法权益，建立农民工工资保障金制度。认真落实各项强农惠农政策，简化各类补贴发放方式，支持中直六安粮库继续执行好国家最低收购价政策，做好大中型水库移民后期扶持资金发放和项目扶持，推进惠民资金管理“一线实”改革。继续实施整村脱贫，脱贫185个村、8.8万人。加强农民负担监管，加大督查和案件查处力度。
　　（三）进一步完善中心城市功能，不断提升城镇化水平。抓住高速公路和高速铁路相继建成以及省会经济圈、新桥国际机场建设的新机遇，立足于加快建成60平方公里、60万人口的中心城市规模，着眼于市区100平方公里、100万人口的规划远景，向东继续推进，向南逐步完善，向西适时展开，向北积极延伸，做大做强中心城市，推动城镇化进程，打造跨越式发展的新平台。
　　加强中心城市建设和管理。坚持拓展框架、完善功能、提升环境、强化管理，做大融资平台，完善中心城市规划体系，提高建设管理水平。抓好高速公路连接线、新区污水管网一期、寿春路、淠望路、淠河总干治理等续建工程，开工刘园路、解放路后续改造等道路畅通工程，实施312沿线、紫竹林路整治、皖西大道开发区段环境综合改造等环境提升工程，推进大雁河治理、城市水系改造等排水排污工程，抓好儿童福利中心等公益设施工程和便民道路、公厕等便民利民工程。启动城市防洪规划修编工作，加强城市防洪工程建设和管理。全面推进社区建设，健全管理和服务体制，把社区建设成为管理有序、服务完善、文明祥和的社会生活共同体。在百日会战的基础上，健全城市管理长效机制，完善市容环境管理责任区，推行数字化城市管理。充分发挥行政执法局职能作用，加强综合执法，加大违法建设拆除力度。大力开展义务植树，提高城市绿化水平。
　　抓好县域中心城镇和小城镇建设。把县城和小城镇建设开发摆上区域发展的战略位置，加大对城镇建设的指导力度，建立激励机制，突出抓好50个重点镇及新农村建设示范镇的建设。壮大县城和中心镇的产业基础，协调推进城镇化和工业化进程，努力把功能做优、环境做美、特色做亮、实力做强。推进城市基础设施与农村对接、城市公共服务向农村延伸，促进城乡一体化。
　　（四）着力引导投资增长，努力扩大消费需求。充分发挥投资和消费对经济增长的促进作用，有效扩大投资和增加消费，进一步增强经济发展的自主性、协调性、持续性。
　　推进重大项目建设。深入实施项目带动战略，全面提升“568”计划。加快推进江汽齿轮45万台乘用车变速箱、华润雪花40万千升啤酒、大昌120万吨球团二期和100万吨球墨铸造、李楼500万吨矿石采选、金安钢铁50万吨合金钢、鸿阳新能源科技园、应流15万吨不锈钢、华源制药液体制剂、索伊电器二期等重大工业项目。加快基础设施项目建设，推动阜六高速公路尽快恢复施工，力促阜六铁路尽快上马，全力支持新桥国际机场建设，实施310省道霍寿段改造；完成白莲崖水库等国家重点治淮工程、行蓄洪区安全建设和移民迁建任务，争取淠河二期治理项目，实施行蓄洪区调整改造、一般堤防加固、平原洼地治理新三项治淮工程，抓好六安港总体规划实施工作；建成500千伏输变电等工程，争取华电二期和金寨抽水蓄能电站项目。认真实施省会经济圈发展规划，推进交通、通讯等基础设施一体化进程。
　　开拓消费市场。巩固和扩大“万村千乡”市场工程成果，继续推进“新网工程”，加快农村现代流通体系建设。加大城市商业网点规划实施力度，积极发展连锁、超市、大卖场等新型流通业态。积极推进商业双进工程，引导连锁企业设立各类网点。合理引导住房、汽车等消费，培育保健、健身等新的消费热点。
　　发展旅游业。完成市旅游发展总体规划修编和红色旅游发展规划编制，实施好旅游发展三年行动计划。加强旅游资源整合，打造大别山和寿县古城特色旅游品牌，建设红绿古特交相辉映的旅游目的地。抓好景区升级、饭店创星工程，完善旅游基础设施建设。大力营造优质旅游环境，广泛组织旅游推介活动，努力扩大旅游市场。
　　（五）进一步深化改革，提升开放型经济水平。统筹对内搞活和对外开放，激发全方位发展新活力，构建多元化开放新格局。
　　深化重点领域改革。推进财税体制改革，建立健全各类国有资产管理体制，强化公共财政职能和国有资产合理配置、有效利用。全面完成市属商贸流通企业改革扫尾，加快市属供销等系统企业改制进度。深化农村综合改革，抓好集体林权制度和水管体制改革。
　　加大招商引资力度。更加积极主动地抓住发达地区产业、资本转移的机遇，坚持靠优良环境、优质资源、优势产业扩大招商。实施产业招商，积极引进带动力强的重大项目和有利于骨干企业、支柱产业成长壮大的项目。积极开展驻外招商、以商招商和专业园区招商，完善“一把手”负责制和目标管理责任制。认真筹备参加国际徽商大会、中国投资贸易洽谈会等重大招商活动。
　　开展全民创业。完善和落实促进全民创业的政策措施，建立健全服务体系，加强创业指导和培训，推进创业基地建设，发展中介服务，营造想创业、能创业、创大业的良好环境。大力实施凤还巢工程，吸引更多有实力、懂技术、会经营的人士回乡创业。进一步促进非公有制经济发展，推进公平准入，改善融资环境，培育更多充满活力的市场主体。
　　改善金融生态环境。巩固政银企合作的良好势头，完善协调机制，保持信贷增长的稳定性和持续性，力争新增贷款50亿元。支持金融机构深入开展“六安发展我发展、我为六安做贡献”活动，推进银行业体系建设，支持农业发展银行拓展商业信贷业务，促进国有商业银行、徽商银行深化改革，鼓励金融机构开展多种方式的服务地方经济创新活动。加快邮政储蓄银行建设，积极扶持农村信用社等地方法人金融机构加快发展，争取市外金融机构在我市开展和扩大信贷业务。加强信用担保体系建设，鼓励保险业、证券业做大做强。
　　扩大进出口规模。坚持以质取胜，大力培育出口品牌，继续推动轻纺及服装、羽绒、丝绸、柳竹制品、机电、建材等产品扩大出口，巩固扩大7个农产品出口示范基地。加大扶持力度，培育进出口超3000万美元企业1家、超千万美元10家，新增进出口企业60家以上。积极组织进出口企业参加广交会、华交会等交易会，鼓励企业境外设立营销公司和生产基地。抓住国家鼓励进口的政策，推动关键技术和设备的进口。
　　（六）切实加强资源节约和环境保护，建设生态文明。坚持把节约资源、保护环境、集约用地作为转变发展方式的重要突破口，确保实现节能减排的约束性目标，推动经济结构优化升级。
　　加强生态市建设。继续推进省生态建设综合示范市及金寨、舒城国家级生态示范区建设，加快霍山国家级生态县创建工作，确保今年通过验收。完善生态市重点工程项目库，加快重点项目建设。继续创建环境优美乡镇，落实好省“百镇千村万户”生态示范工程。做好大别山（六安）世界地质公园申报创建。加强饮用水源保护。开展“三线、两片、一圈”造林绿化，抓好生态公益林建设，推进退耕还林工程，争取国家生物质能源林建设项目。
　　推进节能减排。深入开展全省百家节能行动计划，积极争取和加快实施节能改造及污染治理国债项目，推进新中天、英明化工等重点污染源治理工程，建成五县污水处理厂。发展循环经济，推广清洁生产。继续深入开展环保专项行动，严格新上项目环境影响评价，认真履行“三同时”，加快淘汰落后工艺、设备和生产能力，推动产业结构调整。推进节能减排全民行动，强化非工业领域节能降耗工作，加强环境和节能监察能力建设，做好节能减排统计、监测和考核工作，完善环境公告制度，完成全国第一次污染源普查和土壤污染状况调查。 
　　加强土地节约集约利用。认真开展第二次土地调查，高质量做好土地利用总体规划修编。严格建设用地供应管理，优化土地供应结构，优先保障重点项目建设用地。积极开展土地整理和村庄整理，复垦废弃地，用足用活建设用地置换政策，努力破解土地制约瓶颈。加强资源综合开发管理，严禁各类破坏性开发。

**四、大力推进文化发展繁荣，
提升六安软实力**

　　必须从全面落实科学发展观、构建和谐社会的高度，以建设社会主义核心价值体系为根本，大力加强文化建设，用先进文化引领全市人民奋力推进跨越式发展。
　　加强精神文明建设。认真学习党的十七大精神，深化对科学发展观、构建和谐社会等重大战略思想的宣传教育。把建设社会主义核心价值体系融入国民教育和精神文明建设全过程，用社会主义荣辱观引领风尚，引导全社会进一步树立民主法治、自由平等、公平正义的理念，引导人们自觉履行法定义务、社会责任、家庭责任。加强未成年人思想道德建设，构建学校、家庭、社会“三位一体”的工作网络。广泛开展群众性精神文明创建活动，完成争创全省文明城市三年行动计划，早日实现创建省级文明城市目标。
　　打造地域文化品牌。按照地域性、个性化要求，立足我市独特而丰富的文化资源，强化资源整合，增强六安文化表现力、影响力和竞争力。进一步加强古文化和红色文化的挖掘、开发和利用，做好革命遗址和寿春古城遗址的保护。以双墩一号汉墓发掘保护为契机，挖掘我市汉文化内涵。保护青山绿水，弘扬淳风古韵，发展生态文化和民俗文化。开展第三次文物普查，做好庐剧等国家、省级非物质文化遗产的传承与保护，建立市、县（区）非物质文化遗产名录。
　　发展文化产业。进一步调整结构，优化布局，着力构建以市场为导向、以资本为纽带、政府宏观调控、多种经济形式并存的文化产业发展机制。建设以文化商业为主体的文化大世界项目，加快文化产业基地和区域性特色文化产业群建设，培育产业骨干企业和带头人，壮大文化市场主体，合理引导文化消费，推动艺术产品产业化生产和商品化经营，促进文化产业与旅游、经贸、城建等领域的广泛融合。做大做强现代传媒业，提升报业和广电业综合实力。
　　健全公共文化服务体系。优先建设与群众切身利益紧密相关的文化项目，加快建设市博物馆、全民健身活动中心；加大文化项目争取和市场化运作力度，适时筹建市图书馆、文化馆、报业大厦；以建设综合性、多功能乡镇文化站为龙头，加快农村文化设施建设。继续开展送戏下乡，实施文化信息资源共享、农村电影2131、广播电视村村通、数字电视整体转换、农家书屋等公共服务工程。加大文艺人才培训力度，加强文化队伍建设。落实奖励措施，鼓励创作更多具有皖西地域特色、群众喜闻乐见的优秀文化产品。启动文化事业单位体制改革，加强新闻出版、演出娱乐、网吧等方面管理。强化档案征集管理，做好档案保护工作。

**五、切实解决民生问题，
加快构建和谐六安**

　　按照“学有所教、劳有所得、病有所医、老有所养、住有所居”的要求，以实施省十八项民生工程为抓手，让更多群众得到实惠，让群众得到更多实惠。
　　坚持教育优先发展。进一步提高基础教育工作整体水平，加快新一轮中小学布局调整和农村寄宿制学校建设，消除D级危房22.7万平方米，加强农村教师队伍建设，逐步减少薄弱学校，促进义务教育均衡发展。完善城乡义务教育经费保障机制改革，继续做好农村义务教育“两免一补”工作，加快城区义务教育阶段学校改扩建。高度重视幼儿教育，抓好普通高中教育，扩大优质教育资源。创新职业教育发展模式，壮大职业教育规模。开展贫困家庭高中生资助工作，推动育才关怀行动。规范中小学收费和教材征订行为，加大校园周边环境整治力度。
　　做好就业再就业工作。全面贯彻《就业促进法》，落实和完善积极的就业政策，加强公共就业服务，深入开展对零就业家庭和就业困难人员的就业援助，积极稳妥地做好关闭破产企业职工安置，抓好被征地农民就业，实现再就业1.2万人。全面贯彻《劳动合同法》，提高劳动合同签订率，建立劳动用工备案制度，开展劳动用工监督检查，加强劳动争议处理，构建和谐劳动关系。
　　健全社会保障体系。加大各项社会保险扩面征缴力度，重点推进非公经济组织、城镇灵活就业人员和农民工参加保险，完善被征地农民养老保险制度，提高企业退休人员养老金标准，扩大农村养老保险制度试点，抓好“五保之家”建设。完善城乡居民最低生活保障制度，确保农村低保应保尽保。提高最低工资标准，探索企业职工工资正常增长和支付保障机制，认真落实因基本生活必需品价格上涨对低收入群众的补助办法。加大住房保障支出，健全廉租住房制度，规范经济适用住房建设和管理，加快安置房建设，进一步扩大住房公积金覆盖面。
　　加强卫生服务体系建设。完善新型农村合作医疗制度，基本实现城镇居民医疗保险全覆盖，规范运行机制，提高管理水平。巩固乡村卫生服务一体化管理体制，改建39所标准化乡镇卫生院、290所标准化村卫生室。提高社区卫生服务能力，新建29所城市社区卫生服务机构。充分发挥现有卫生机构服务作用，建立疾控机构与医疗、保健等机构协作机制，构建城乡卫生安全网。支持城区重点医院发展和六安卫校申报安徽卫生职业学院。进一步做好艾滋病、结核病等重大传染病防治工作。
　　完善社会管理。建立健全计划生育“村为主”、性别比综合治理、社会抚养费征管三大工作机制，完善农村计划生育家庭奖励扶助制度，继续稳定低生育水平，提高出生人口素质。围绕把六安建成全省最稳定的市之一，加强“平安六安”建设，完善社会治安防控体系，确保“三基”工程圆满完成，支持霍山县力夺全国社会治安综合治理“长安杯”。深入开展矛盾纠纷排查调处，妥善解决信访群众的合理诉求，进一步规范信访工作和群众信访行为。加强价格监测，确保物价指数涨幅控制在省平均水平以内。整顿和规范市场秩序，强化食品、药品、餐饮卫生监管。严格落实安全生产责任制，加强基层基础工作，加大重点行业、重点领域专项整治力度，杜绝特大恶性事故发生。巩固和发展平等团结互助和谐的民族关系，依法加强对宗教事务的管理。深入开展“五五”普法，扩大法律援助覆盖面，加大维权救助力度。认真抓好青少年阳光体育运动，推进迎奥运、促健身活动，办好市第二届运动会。提高统计基层基础工作水平，做好第二次全国经济普查。巩固和扩大基层民主，做好村民委员会换届工作，支持工会、共青团、妇联等群众团体独立自主地开展工作。重视社会组织建设和管理，关心老龄、妇女儿童工作，发展残疾人事业，推进外事、侨务和对台工作，做好地震、气象、地方志等工作。巩固创建“全国双拥模范城”成果，做好优抚工作，支持驻地部队建设，加强后备力量建设和民防工作，提高国防动员和民兵预备役工作水平。

**六、加强政府自身建设，
努力建设人民满意政府**

　　新的形势，新的任务，对政府建设提出了新的更高要求。我们必须增强加快发展的紧迫感、时不我待的机遇感、勇挑重担的责任感、不负众望的使命感，建设勇于创新、求真务实、勤政为民、廉洁高效的政府。
　　转变职能，增强服务力。进一步强化服务理念，把经济管理的主要精力放到为市场主体服务上来，努力创造公平竞争的市场环境、稳定透明的政策环境、优质高效的服务环境。加强社会管理和公共服务职能，把财力物力更多地集中到公共服务上来，把政策着力点更多地放到解决民生问题上来。提升市政务服务中心功能，加快“两集中、三到位”改革，促进市、县、乡镇三级中心联动，打造全省一流的行政服务平台。建立健全反应灵敏、协调有序、运转高效的预警和应急机制，加强应急培训和演练，提高应对突发事件能力。
　　依法行政，增强公信力。坚持把依法行政贯穿于政府决策、执行、监督的全过程。推进政务公开和决策科学化民主化，完善集体决策、专家论证、重大事项社会公示、听证等制度。强化行政执法培训，认真落实行政问责制、行政执法责任制和过错责任追究制，做好行政复议和行政应诉，坚决查处、纠正违法和不当的行政行为。进一步转变执法理念，促进在合法行政基础上合理行政，规范行政执法自由裁量权。推进相对集中行政处罚权，切实解决多头执法、重复执法、执法扰企、执法扰民问题。各级政府要更加自觉接受人大、政协监督，主动向人大报告工作，认真执行人大及其常委会的决议、决定，认真办理人大议案、代表建议和政协建议案、委员提案，主动把政治协商纳入决策程序，诚恳听取各民主党派、工商联、无党派人士及各人民团体的建议和意见，鼓励支持人民群众和新闻媒体的监督。
　　提升效能，增强执行力。进一步理顺职能分工，优化工作流程，形成规范有序、公正透明、高效便捷的运行机制。扎实开展“三型机关”创建活动，推行全程代办制、服务承诺制、首办负责制、限时办结制和超时默认制。创新行政服务和管理方式，发展电子政务，推进在线服务、网上审批。加强政风行风建设，强化效能监察，发挥政府网站、市长热线、政风行风热线等平台作用。深入开展“发展环境暨政风行风万人评”活动，把评判权、监督权交给群众和服务对象。加强经济发展环境案件投诉办理，严肃查处影响和破坏环境的人和事。各级政府及其部门的领导同志要深入基层、深入群众、深入实际，到条件差、矛盾大、困难多的地方，调查研究，破解难题，服务群众，推动发展。
　　廉洁从政，增强约束力。坚持源头治理，规范行政审批和政府投资项目管理，完善招投标运作机制，严格规范建设工程招投标、国土资源和产权交易、政府采购等行为，推进科学合理的招投标交易和监管方式，加强行政收费监管，加强领导干部经济责任审计，试行审计公告制度。认真执行公务接待制度，压缩会议费、招待费等一般性支出，降低行政成本。坚决查处违法违纪案件，切实纠正损害群众利益的不正之风。认真落实党风廉政建设责任制和责任追究制，突出抓好各级领导干部的廉洁自律，进一步树立干净干事、正气硬气的公务员形象，进一步树立勤政廉政、亲民为民的政府形象。
　　各位代表！党的十七大提出了全面建设小康社会的新要求，掀开了我国社会主义现代化建设的新篇章，六安也步入了奋力崛起、加速跨越的新征程。让我们在中共六安市委的坚强领导下，高举中国特色社会主义伟大旗帜，同心同德，开拓进取，加快实现“三大跨越”、“三个翻番”，向着夺取全面建设小康六安新胜利的目标奋勇前进！
